# The value of donkeys to livelihood provision in northern Ghana

**DOI:** 10.1371/journal.pone.0274337

**Published:** 2023-02-22

**Authors:** Heather C. Maggs, Andrew Ainslie, Richard M. Bennett

**Affiliations:** School of Agriculture, Policy and Development, University of Reading, Reading, Berkshire, United Kingdom; Szechenyi Istvan University: Szechenyi Istvan Egyetem, HUNGARY

## Abstract

Increased demand for the supply of donkey hides for use in the Traditional Chinese Medicine e’jiao, is leading to a re-appraisal of donkeys’ contributions to livelihoods across the world. This research aimed to understand the utilitarian value donkeys provide to poor small holder farmers, especially women, in their efforts to make a living in two rural communities in northern Ghana. Uniquely, children and donkey butchers were interviewed for the first time about their donkeys. A qualitative thematic analysis was undertaken of data disaggregated by sex, age and donkey-ownership. The majority of protocols were repeated during a second visit, ensuring comparative data between one wet, and one dry season. Donkeys are more important in people’s lives than had previously been recognised and are highly valued by their owners for their help in reducing drudgery and the multi-functional services they offer. Hiring out donkeys to generate income is a secondary role for people who own donkeys, especially women. However, for financial and cultural reasons the way donkeys are kept results in the loss of a certain percentage of the animals to the donkey meat market, as well as the global hides trade. Increasing demand for donkey meat, coupled with increasing demand for donkeys for farming, is leading to donkey price inflation and theft of donkeys. This is putting pressure on the donkey population of neighbouring Burkina Faso and pricing resource-poor non-donkey owners out of the market. E’jiao has put the spotlight on the value of dead donkeys for the first time, especially to governments and middlemen. This study shows that the value of live donkeys to poor farming households is substantial. It attempts to understand and document this value thoroughly, should the majority of donkeys in West Africa be rounded up and slaughtered for the value of their meat and skin instead.

## Introduction

Donkeys were once status symbols [2], lauded as the drivers of the development of ancient civilizations [3, 4]. As the first domesticated transport animal [3] donkeys have been used by humans for millennia for draught animal power. There is no one literature relating to donkeys. Academic papers relating to donkeys include intermediate transport [5–7], draught animal power [8–10], the clinical veterinary, health and welfare disciplines [11–14] and, increasingly donkey commodities [15–19]. There are also a number of papers specifically about donkeys in Ghana, including transport [20], draught animal power [21, 22], gender [5, 7, 10], livelihoods [10] and welfare [23]. However, most papers do not specify the core intrinsic or extrinsic value of donkeys from other elements of their instrumental value, including power provision and portage for example [5] or power provision and livelihoods [20, 23]. A further section of papers is not donkey specific, but have a different focus, for example on draught animal power or working equids [24–26]. These papers include information on donkeys only if data was collected about them—within a livestock assessment for example.

However, the use of donkeys in a Traditional Chinese Medicine (TCM) called e’jiao has also taken place across millennia [16, 27]. E’jiao’s constituent ingredient is extracted from boiling donkey skins in water. Originally reserved for the use of the emperor and the wealthy, demand for e’jiao has increased steeply since the 1990s, due to the growth in Chinese disposable income and clever promotion of both TCM and e’jiao [16, 27]. E’jiao is now a “coveted luxury product for ostentatious consumption” [27:1], with new indications for use and astute product development contributing to increasing demand [16, 27]. E’jiao manufacturers have reported they need between four to ten million donkey skins a year [27], outstripping the ability of China to meet demand from its own donkey population, which stood at ~2.3m in 2020m [28]. This requirement has led to a corresponding increase in the demand for donkey hides from around the world, resulting in an increase in the price of e’jiao, donkeys and donkey hides. As Bennett and Pfuderer [16:3] make clear the e’jiao industry “has far reaching global implications for donkey populations.”

A 2017 report entitled “Under the Skin,” by a UK charitable non-governmental organisation, the Donkey Sanctuary [29], brought these issues to a much wider, global audience. This report resulted from the increasing awareness amongst the Sanctuary’s network of global partners of the potential impacts the donkey hide trade could have on donkey populations and their welfare. The report established the issues facing donkeys and their owners/users as important problems to be addressed. It has also initiated interest in donkeys from academia, development agencies and international NGOs [1, 30, 31], signalling a change in the direction of the traditional narrative of the valueless donkey.

Bough [3] argues that the domesticated donkey lost its reputation and status as ancient trade routes facilitated the spread of donkeys to the Near East, the Mediterranean, India and China. Thus, donkeys have become associated with poor people [7, 13, 30, 32], who don’t tend to write history. While a majority of studies prior to 2017 conclude that donkeys have little value through many different interpretations and nuances of the word [31], there are a number of papers (i) highlighting how donkeys can reduce drudgery [30, 33–35]; (ii) relating to donkeys being hired out for income [34–37] and (iii) on the advantages donkeys have for use by women [7, 20, 25, 30, 35]. These references primarily relate donkeys’ value to the extrinsic “economic utility” they provide humans [38:2]. The concept of utility has been integral in theories of value in classical, neo-classical and neo-liberal economics since the 18^th^ century, although its usefulness is contested [39, 40]. Thus, the near universal narrative of the valueless, invisible donkey [30, 31], seen as a “poverty indicator, rather than an agent of change” [30: 243], led to the embedded perception of donkeys as having little intrinsic or extrinsic value. This traditional view is beginning to change in the context of the new international forces supplying donkey hides for use in the manufacture of e’jiao. The commodification of donkeys and donkey hides to supply the e’jiao market also provides a utilitarian context to this research.

**Fig 1:**
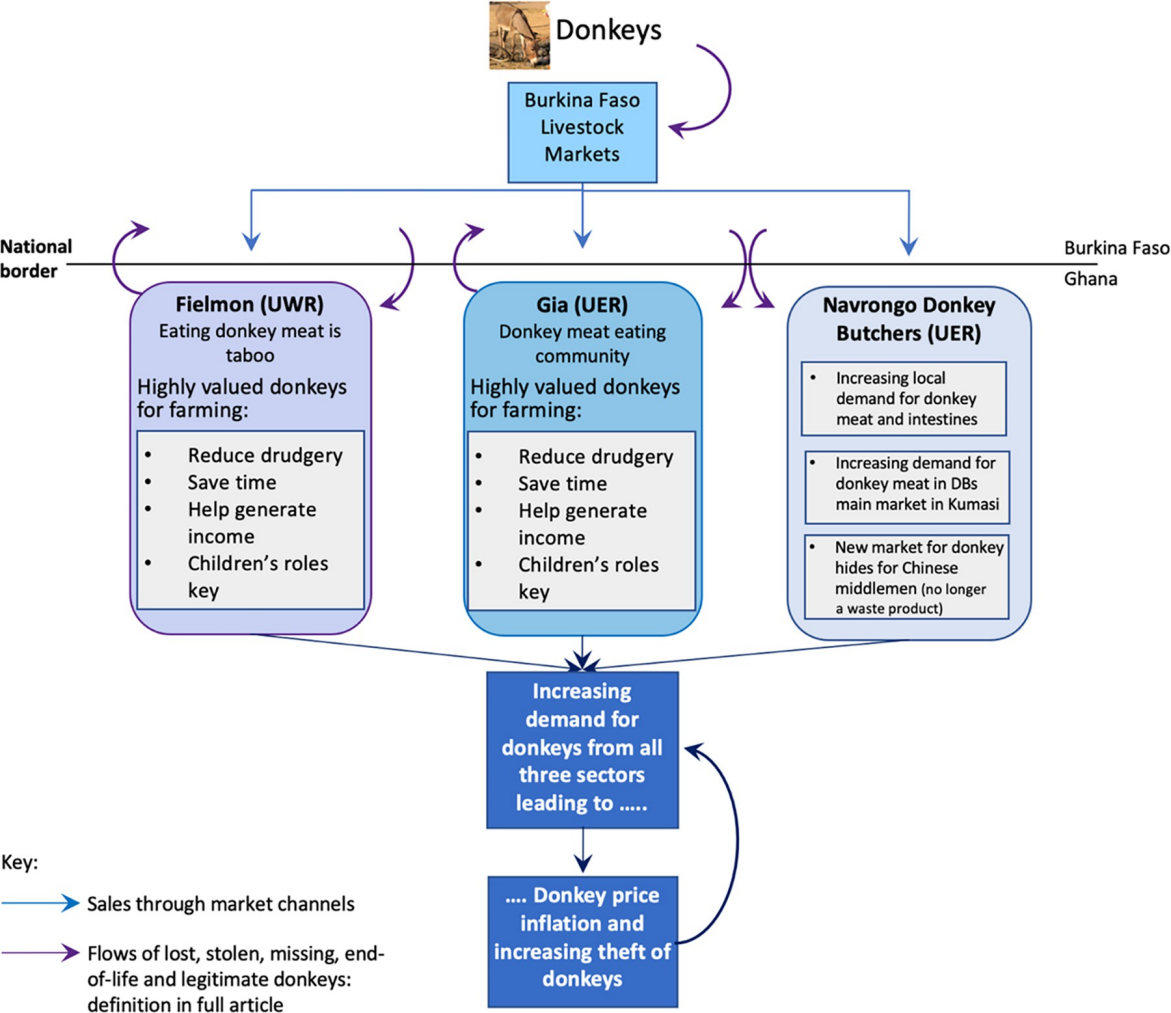
Graphical abstract. (Source: Maggs, 2022 [1]).

E’jiao has focused a spotlight on the value of dead donkeys, to both governments and middlemen. This is leading to a reappraisal of the usefulness of live donkeys to people labelled as poor [41], because unless worked, donkeys are rarely owned and maintained [42]. This research takes a utilitarian approach to the value of donkeys in answering the aim of this study, which was to examine the role of donkeys in rural households in northern Ghana and their contribution to livelihood outcomes. This paper argues that to the individuals in northern Ghana who depend on them for their livelihood, donkeys, both alive and dead, are highly valuable even if not of particular significance to Ghana’s economy overall (Fig 1).

Evidence for the demand for donkey hides for e’jiao was obtained from the study site in Gia, which is opportunistically riding on the back of the pre-existing and growing donkey meat market in Ghana. The demand for donkeys for their skins threatens the livelihoods of many poor, rural communities across northern Ghana and indeed the rest of Africa [15–19, 27, 29], who need live donkeys to provide the power to reduce their drudgery and save them time. Section two reviews our materials and methods. Key results and their implications are presented in section three, under five headings: i) donkeys contribution to reducing drudgery; 2) donkeys and income generation iii) the increasing value of donkeys; iv) increasing demand for donkeys and v) missing donkeys: lost or stolen? Concluding remarks are in section four.

## Materials and methods

### Ethics approval

The study’s protocol for researching with children was approved by the University of Reading’s Research Ethics Committee (Ref: UREC 18/52, December 2018 and Ref: UREC 19/44 April 2019). The research instruments for the adult element of the study were approved by the University of Reading’s School of Agriculture, Policy and Development ethics committee (Ref:00809P, November 2018 and Ref: 001026, April 2019). The same assent (child) and consent (adult) protocols were followed across field visits, with no child taking part in multiple research instruments. Copies of all research instruments are included in the data supporting this paper, available at: https://doi.org/10.17864/1947000408.

### Study design

**Fig 2:**
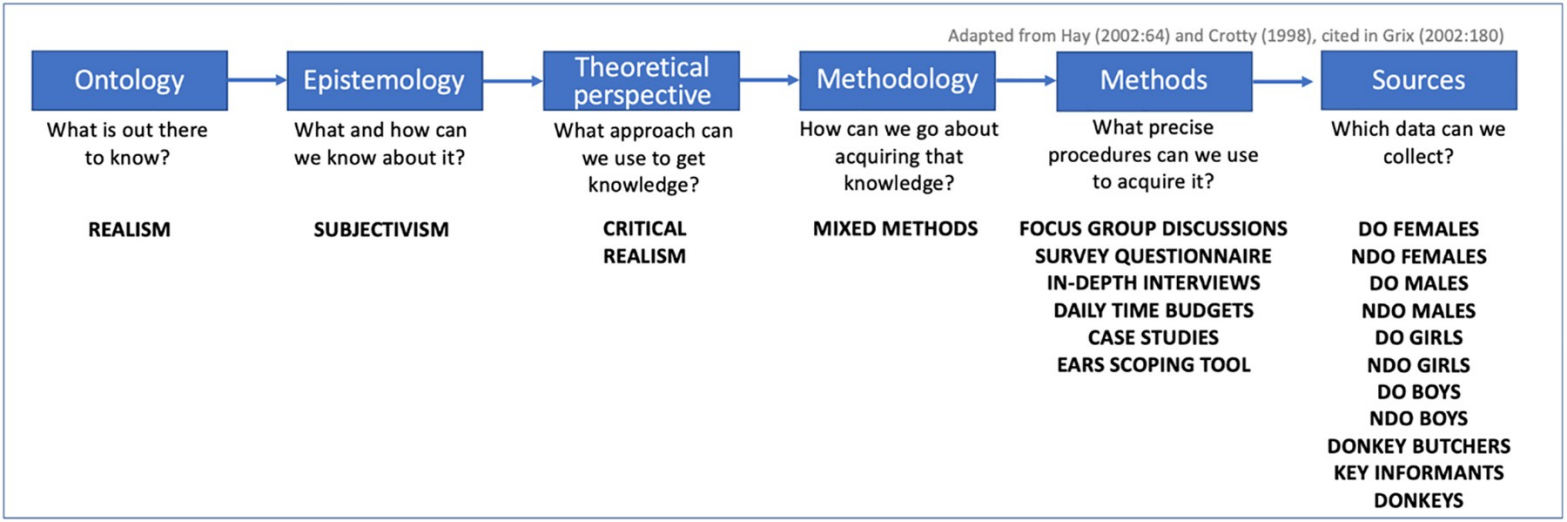
Summary of methodological relationships for this research. Source: adapted from Hay, (2002) and Crotty (1998), cited in Grix [43:180].

The critical realist theoretical perspective of this research and its underpinning philosophy are outlined in diagrammatic form (Fig 2). Grounded in mixed methods, the exploratory research design involved the collection of data at the household level to answer the research question: what relationship do donkeys play in the provision of livelihoods in Ghana?

Twenty-three donkey owners (DO) from Fielmon and five from Gia, plus five non-donkey owning (NDO) respondents from Fielmon and three from Gia completed a survey questionnaire (n = 36). Different DOs (six) and NDOs (four) were interviewed using in-depth, semi-structured questions. The DO in-depth interviewees, who were interviewed to build the donkey owning case studies, completed daily time budgets (Table 1). These budgets were undertaken on seven consecutive evenings to record the household’s use of the family donkey, disaggregated by age and gender. An Equid Assessment Research and Scoping (EARS) Tool evaluation was undertaken with all DOs, across all research instruments [44]. This included questions for owners on the services provided by their donkeys, aspects of the animals’ health and welfare and a welfare assessment of every donkey. Information was also obtained from a range of 10–16 year old children from DO and NDO households, in each of the study sites (Table 1). The ages of all adult respondents ranged from 23 to 67 years old; most were between 31 and 50 years (SD 10.73). The majority of adult respondents were female (61%); over half (61%) had no formal education and all heads of households, as reported by both male and female respondents, were male.

**Table 1:** Study design. Source: Maggs, 2022 [1].

	Fielmon (UWR)			Gia (UER)		
	December 2018	June 2019		December 2018	June 2019	
**FGD (DOs only)**						
Female FGD	1	1		1	1	
Male FGD	1	1		1	1	
Children’s FGD						
Mixed sex pilot	1			1		
Girls only		1			1	
Boys only		1			1	
**Survey Questionnaires**						
Female DOs	14	14		3	3	
Male DOs	9	9		2	2	
Female NDOs	3	3		2	2	
Male NDOs	2	2		1	1	
**In-depth individual interviews**						
Female DOs	2	2	+ 7DTB	2	2	+ 7DTB
Male DOs	1	1	+ 7DTB	1	1	+ 7DTB
Female NDOs	1	1		1	1	
Male NDOs	1	1		1	1	
DO boy and girl child siblings					1	
NDO boy and girl child siblings					1	
DO girl-only interviews		2			2	
NDO girl-only interviews		2			2	
DO boy-only interviews		2			2	
NDO boy-only interviews		2			2	
**Contextual interviews**						
Donkey butchers				4	1	
Veterinary surgeons		2				
Livestock market traders		1			1	
Community elders		2			1	
UDS academic		1				

**DO**: from donkey owning family **NDO**: from non-donkey owning family

**FGD**: Focus group discussions **7DTB**: seven daily time budgets

The six children’s focus group discussions were comprised of 54 DO children (22 girls and 32 boys) and 10 DO children and 10 NDO children were interviewed, comprised of 5 girls and 5 boys each. All were between the ages of 9–16 years-old (SD 1.83). The methods used to collect data from the adult participants (Table 1) were repeated with the same respondents and mostly the same donkeys six months later, after gaining additional funding for a second field visit. All research costs were funded by The Donkey Sanctuary, UK. Purposive and snowball sampling was undertaken to recruit all respondents, except the sibling interviews, where chance was used to recruit the children. All names used in this paper are pseudonyms to aid anonymity. Methods of triangulating our results included: i) specifically asking male and female respondents similar questions in different ways, supported by the seven daily time budgets; ii) returning to the field during the wet season; iii) comparing findings between adult and child respondents and iv) interviewing NDOs to add granularity to the analysis.

### Data collection and location

**Fig 3:**
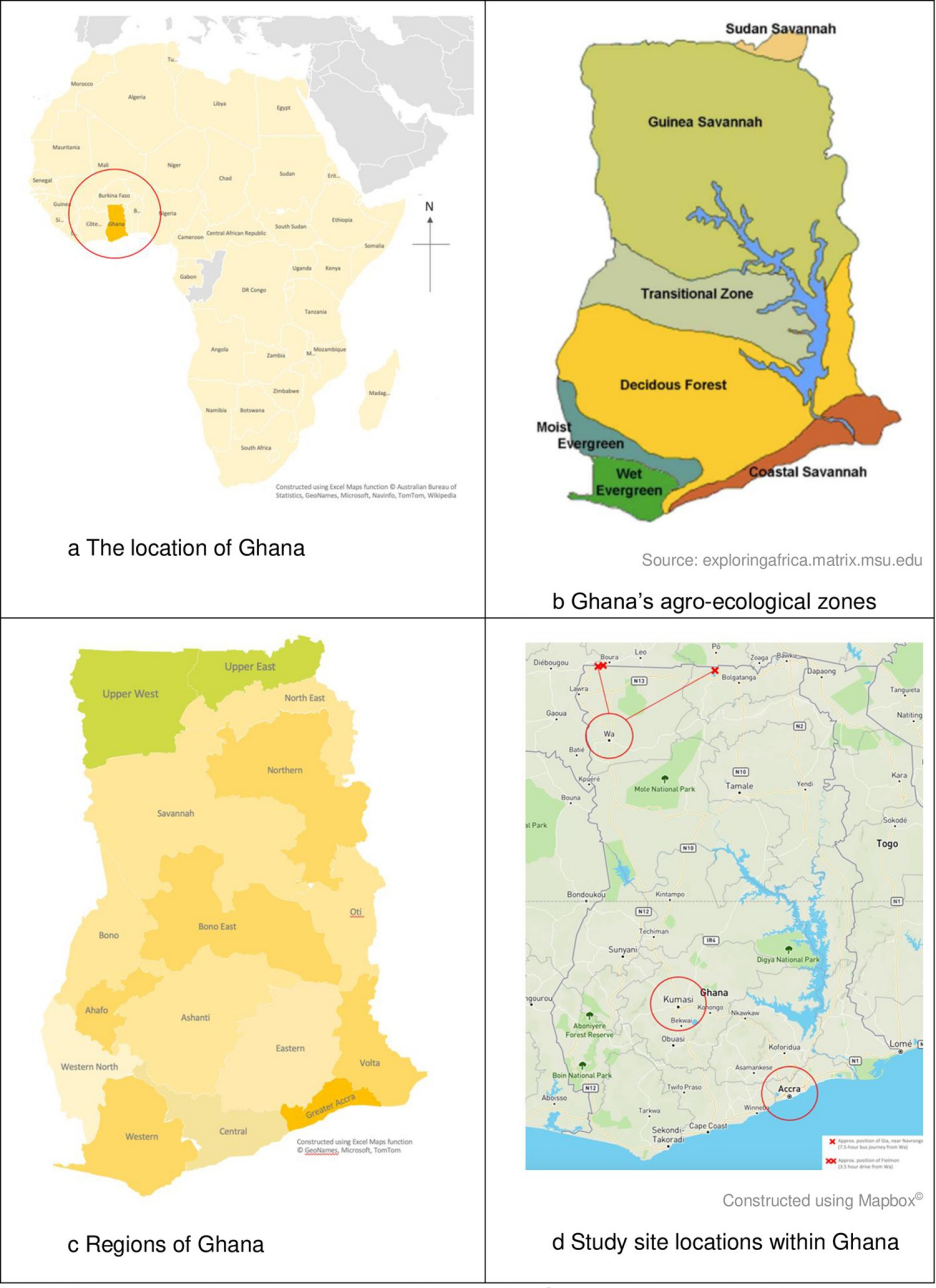
The research environment of this study. Source: Maggs, 2022 [1].

Five key reasons informed the decision to locate fieldwork in northern Ghana: (i) the excellent working relationship between the University of Reading and the University for Development Studies, resulting in innovative research outputs; (ii) the provision by the University for Development Studies of highly qualified and skilled research assistants to help address language barriers in the study sites and ease concerns about the safety of a single female researcher; (iii) confirmation that rural communities do use donkeys to farm in northern Ghana, via pilot focus group discussions; (iv) Ghana has one of the highest number of relevant academic papers published in comparison to alternatives; (v) references to Ghanaian donkeys supplying the global trade in donkey hides had been made in local media accounts and vi) the government of Ghana had placed restrictions on both the slaughter of donkeys and the export of donkey skins in 2017 [29]. Data were primarily collected for this study in two agro-pastoralist communities in northern Ghana: Fielmon in Upper West Region and Gia in Upper East Region, near the Burkina Faso border (Fig 3). Additional interviews with donkey butchers were undertaken in Doba, a community ~8kmEast of Navrongo. Key informants included two veterinary surgeons, village elders and donkey market officials in Burkina Faso.

Two teams of multi-lingual, experienced enumerators were recruited from the UDS for each study site. These research assistants were trained in the project’s aims and objectives by the lead researcher and first author. Their valuable contributions meant all research could be conducted in the local languages of the two communities. Data collection took place across two field visits: the first between November 2018 and January 2019 during the dry season; the second during the wet season in June 2019.

### Analysis

With a critical realist ontological approach and a relational epistemology, a reflexive, qualitative thematic analysis [45] of the data was undertaken in NVivo 12.6.0 for Mac. This meets the Standards for Reporting Qualitative Research [46]. Units of meaning were coded at least twice across all research instruments and were chosen to “honor the participant’s voice … [and] … deepen […] understanding of cultures and worldviews” [47:71]. Final categories and sub-categories were developed from the initial codes and subcodes with reference to Saldaña’s 2015 [47:13] streamlined codes-to-theory model for qualitative inquiry. New insights were co-created with the research participants into the demographics, infrastructure, households and livelihood practices of both DOs and NDOs.

### Limitations

The key limitations of this study relate to bias and the self-reported nature of the data collected. Some of the participants may have answered carelessly [48], or deliberately or unconsciously provided misleading responses. The data may also be subject by social desirability bias and acquiescence bias, especially because of the presence of a WEIRD (from a white, educated, industrialised, rich and developed country [49]) female researcher. Some respondents may have been reluctant to reveal accurate information about sensitive issues, for example their income/expenditure. These respondents may have provided misinformation, or else they may have provided information that they thought would conform to their social norms. However, it must be noted that like most psychological research the evidence base for assertions of bias have primarily been undertaken in WEIRD societies, not in the Majority World [49, 50]. A reflexive thematic analysis was undertaken on the collected data to take into account researcher bias, another potential threat to the validity of the findings of this study. To minimise this occurring, the research was undertaken with all the participants and respondents, rather than to, or for them [51].

## Results

### Introduction

**Fig 4:**
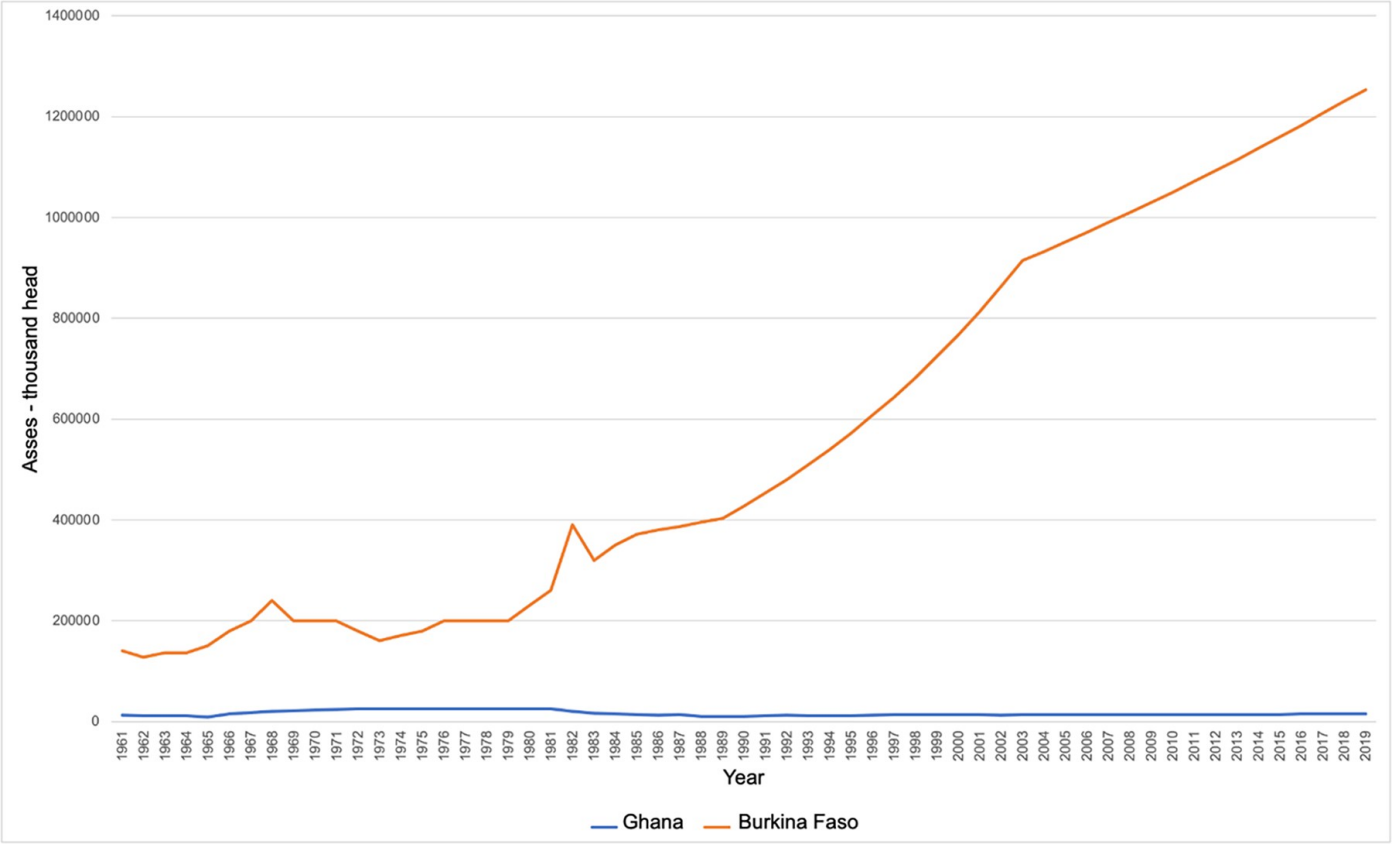
Donkey populations of Ghana and Burkina Faso 1961–2019. Notes: the quality of the data differs, with population estimates for both countries based on official data, FAO estimates and FAO data based on imputation methodology. FAO. Source: FAOSTAT/asses [28].

Unlike southern Ghana, which has heavier soils and two wet seasons annually, northern Ghana has only one wet season a year. Coupled with sandy, light soils, which facilitate the use of donkeys [35], these conditions prohibit the cultivation of cocoa in the North, Ghana’s chief agricultural export [35, 52]. The major economic activity in this region of Ghana is therefore small-scale crop, livestock, and poultry production using traditional methods, with little modernization and mechanization. Ghana has ~14,910 donkeys [28], concentrated within its three northernmost regions. These regions are all close to the border with Burkina Faso, which in comparison has ~1.25 million donkeys (Fig 4). According to official figures, Ghana’s donkey population has remained stable for the last fifty years, whereas the donkey population of Burkina Faso has been steadily increasing over the same time period. This could indicate an increase in the numbers of poor people requiring their services [42]. The proximity of the Burkina Faso border to the agro-pastoralist communities where data was collected for this research provides important context to the study’s findings.

Data were collected from three different groups of users of donkeys: those who owned them for domestic and agricultural assistance who taboo the eating of donkey meat (Fielmon, in Upper West Region); those who own them for similar reasons but are increasingly eating donkey meat (Gia in Upper East Region, 191km to the East of Fielmon) and those who purchase them to slaughter as soon as possible, making their living from the meat and hides trade (Gia and Doba, Upper East Region). For effective assistance with income generation farming and domestic activities a donkey on its own is not enough. DOs need a cart and/or plough, which are essential and require additional financial outlay [7]. A minimum of two donkeys are needed for farming to fit the region’s plough and implement designs, unless DOs harness their one donkey to a cow if owned, or beg, borrow or hire another animal when needed. One household reported that they owned a cart but were saving up for the donkey.

### Reduction in drudgery

**Fig 5:**
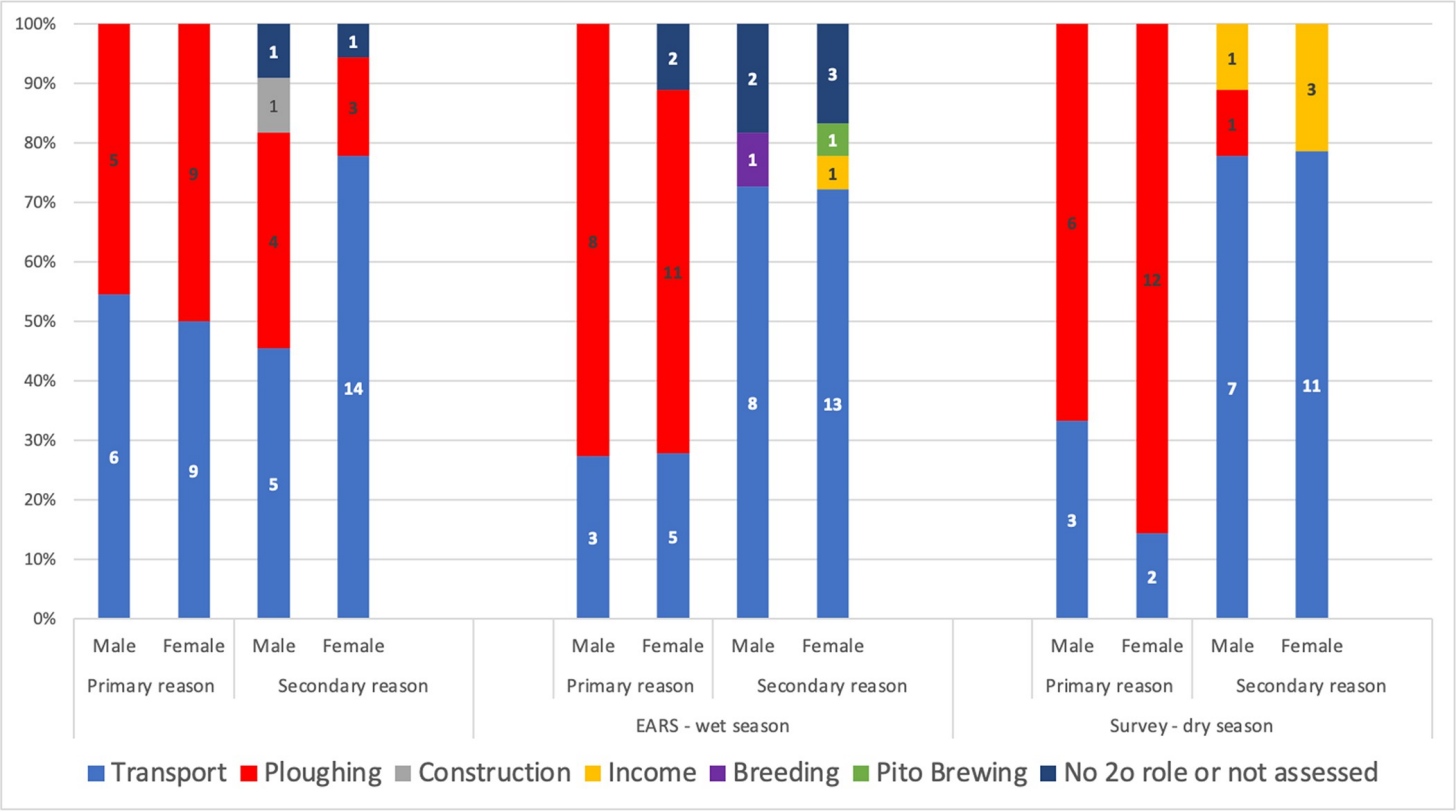
All-source reasons why people keep donkeys in northern Ghana, split by gender (n = 29). Source: Maggs, 2022 [1].

Not surprisingly, the key reasons agro-pastoralist farmers in northern Ghana gave for owning a donkey in our questionnaire and EARS survey included ploughing and transporting a diversity of goods. These reasons differed depending on study site and season (Table 2 and Fig 5). Ploughing and farming activities were more important to Fielmon DOs across all seasons. In this community the eating of donkey meat is tabooed. Because of a lack of irrigation they rely on rain-fed and compound farming–a type of farming system involving livestock and agricultural crops within or near household compounds. However, Fielmon does have a main market in the community. The key reason Gia DOs gave for owning donkeys was transportation. However, this community has access to irrigation from the Tono Dam and rely on gardening and compound farming for their livelihoods. Their produce, including chillies, peppers and tomatoes, is perishable to a greater degree than the subsistence crops grown in Fielmon and need time-sensitive transportation to Navrongo market 6.5km away. The consumption of donkey meat, including donkey intestine soup, is increasingly fashionable in the Upper East Region where Gia and Doba are located [31].

**Table 2:** Frequencies of key donkey tasks/activities per month. Source: Maggs, 2022, survey data [1].

Key Task/ Activities		Months												Frequency	
		M	J	J	A	S	O	N	D	J	F	M	A	Total	%
Transporting/Carting	**Food items/produce** ^a^	**2**	**1**	**0**	**1**	**15**	**23**	**23**	**3**	**1**	**0**	**0**	**0**	**69**	**19.22%**
	**Firewood**	**8**	**4**	**2**	**2**	**1**	**1**	**0**	**12**	**17**	**7**	**5**	**4**	**63**	**17.54%**
	**Water**	**6**	**0**	**1**	**0**	**1**	**0**	**7**	**8**	**4**	**9**	**8**	**6**	**50**	**14%**
	Building materials^b^	3	1	0	0	0	0	1	4	6	5	2	1	23	6.4%
	Manure/Fertiliser	1	3	0	2	0	0	1	1	3	3	3	6	23	6.4%
	People	2	1	1	1	0	0	0	0	0	0	2	4	11	3.0%
	Charcoal	0	0	0	0	0	0	0	0	1	0	1	2	4	1.1%
	Animal feed	0	0	0	0	0	0	0	1	1	0	0	0	2	0.55%
	Seedlings	0	0	0	0	0	0	0	0	0	0	1	1	2	0.55%
	Sub-total													247	68.8%
Agriculture	**Ploughing**	**8**	**17**	**13**	**0**	**0**	**0**	**0**	**0**	**0**	**0**	**0**	**1**	**39**	**10.86%**
	Earthing up/out	0	0	1	12	0	0	0	0	0	0	0	0	13	3.62%
	Sowing beans	0	0	2	0	0	0	0	0	0	0	0	0	2	0.55%
	Applying herbicide	0	0	0	1	0	0	0	0	0	0	0	0	1	0.27%
	Sub-total													55	15.32%
Other	Going to/from market	2	1	0	0	0	0	1	1	1	3	2	1	12	3.3%
	**Services (for hire)**	**0**	**0**	**0**	**0**	**0**	**0**	**2**	**1**	**0**	**3**	**2**	**1**	**9**	**2.5%**
	Towing cart	0	0	0	1	0	1	0	0	0	0	0	0	2	0.55%
	Sub-total													23	6.4%
None	**At rest/roaming**	**3**	**1**	**3**	**5**	**6**	**1**	**0**	**1**	**0**	**2**	**7**	**5**	**34**	**9.47%**
Grand Total														359	99.99%

^a^Beans, groundnuts, maize, millet, vegetables.

^b^Sand, blocks, bricks.

When asked what activities respondents undertake with their donkey across the year frequencies reveal that the annual activity of ploughing has an annual frequency of nine per cent, compared to seventy per cent for the near-daily task of transportation (Table 2). These include the inherent daily domestic drudgery for women and children all year round. For men, this includes seasonal farming activities, like ploughing and sowing, illuminated by this quote by a male focus group participant (RID52):

“With only one donkey I will never want to sell it at all. This is because the donkey is now my hoe or plough. I can’t do any meaningful farming without it. Why will I sell it?”

However, many men also use their donkey to generate income throughout the year [31].

Gia respondents reported a larger range of alternative activities with their donkey than those from Fielmon. When asked about the secondary reasons why respondents owned donkeys, a number of DOs stated their donkey had no secondary role. However, gender differences are discernible in the reporting of some secondary roles, although the numbers are very small. For example, construction and breeding were both mentioned as secondary reasons by one male respondent each. This equated to 3.45% for breeding, similar to the rate of five per cent found in Tuaruka and Agbolosu’s 2019 study in Ghana’s Northern Region [53]. For Kahiau, the one male in-depth interviewee (RID27) from Gia who mentioned it, “breeding” involved ensuring he had female donkeys to reproduce and keep his re-purchase costs to a minimum:

“If I get money I will buy a female donkey and add to the number I have already, […] because the female donkey gives birth, and it gives birth every year.”

Brewing pito, the local, malt-based beer is exclusively a female domain (Fig 5). Only five respondents stated that the secondary role of their donkey was to generate income and of those five, four were women. Interestingly half of the female respondents from Fielmon stated ploughing as the key reason they owned a donkey, an activity that they don’t physically undertake, as they are culturally discouraged from doing so.

From the survey data, the majority of DO respondents stated they could not manage without a donkey. The majority of NDO respondents (7/8) aspire to donkey ownership with one male saying he didn’t need donkeys as he owns oxen. Many DOs used the word beg when recalling life before the advent of their donkey, as not all DOs have always owned them, as these focus group quotes indicate:

“If you don’t have one and want to build, you have to go and beg or borrow someone’s donkey to work” (RID52/male).

“Who would help you fetch the water? If you beg someone she may not come because she is used to using the donkeys in carrying water” (RID50/female).

There was one hundred per cent agreement to the questions “Do you think donkeys reduce the work of men, women and children?” by DOs and NDOs alike, who also agreed that the saved time and additional transport costs their donkey was the equivalent of saving them ‘money,’ illuminated by this quote from Adwin, a male focus group participant (RID53):

“Donkeys actually give me money than saving me time. I use my donkey to transport vegetable leaves, tomatoes, pepper, and firewood to the market to sell and make money. My donkey also saves me transport cost and that adds me more money to my saving. That is to say the money I would have incurred to transport my items is saved as part of my money. My donkey also saves me and my family time and we used that time again to make money by working on the farm.”

NDOs were interviewed to provide context. Those that cannot afford to hire the services of a donkey and cart are reduced primarily to head loading. This is illustrated by Selma (RID38), a non-donkey owning interviewee from Fielmon, when advising how she has to undertake some of her livelihood chores without draught power (Table 3).

**Table 3:** How one non-donkey owning family undertake key daily tasks. Source: Maggs, 2022 [1].

Daily Chores	How are these chores performed in your household?
Carrying firewood	I mostly harvest them in the farm and then hire the services of the donkeys from the owners to cart them home.
Carrying farm produce	[My husband] uses a bicycle to cart them home while the children and me use baskets and basins to carry some home.
Carrying water	The children use basins to carry the water on their heads home.
Carrying manure	Since we don’t have the donkey to carry them, we mostly apply the manure to the farm by the house [compound farm]. This is because we cannot carry them far. My husband and children mostly spread the manure on the farm. We mostly carry them on our head to the farm to spread.
Carrying pito or other goods to the market	I and my daughter always carry them on our heads to the market.
Ploughing	One of our sons is in secondary school. He normally comes to help in the farming. We use hoes to plough. All the members of the household take part in this activity.
Carrying building materials	My children and I usually carry them on our head.
Transporting people	Bicycles are used for transportation. My husband and I have bicycles we use but the children walk to wherever they are going.

Based on an in-depth case study interview with Selma, a female NDO from Fielmon (RID38)

### Donkeys and income generation

**Fig 6:**
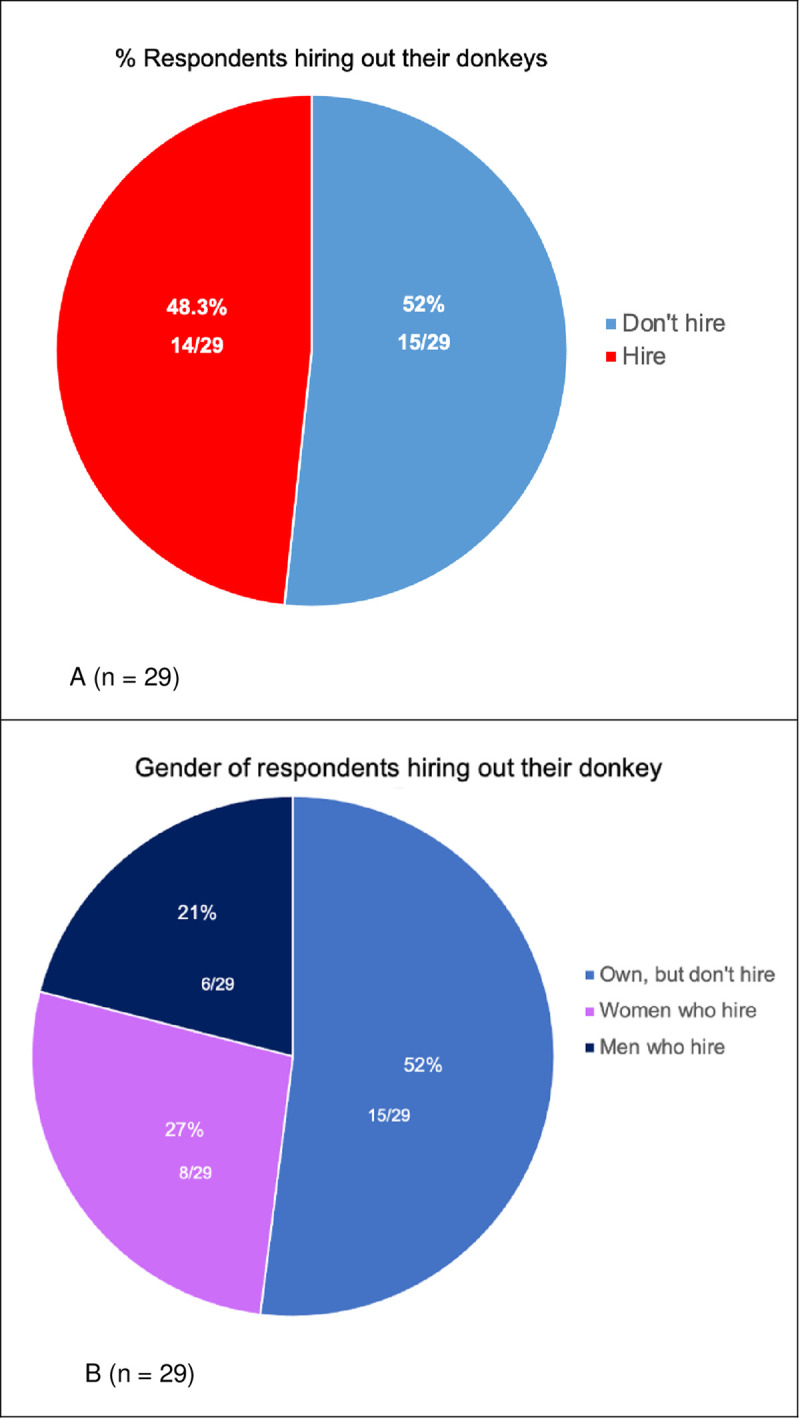
Hiring donkeys out for income. Source: Maggs, 2022 [1].

From the questionnaire, hiring out of donkeys for a fee had an annual frequency of only 2.6%: the lowest of any activity except towing a cart (Table 2). However, triangulating the data with the results from the EARS survey, respondents revealed that other products were carried when their donkeys were hired out, though this was not mentioned in the questionnaire by the same respondents. Nearly fifty per cent (48.3%) of all DOs do actually hire out their donkeys to generate income (Fig 6A). This is especially true for women: only nineteen per cent of female DOs report hiring out their donkey in the survey but of those forty eight per cent hiring out their donkeys, twenty seven per cent were women compared with twenty one per cent men (Fig 6B).

Rich, thick thematic analysis provided further details of additional methods of generating income using a donkey and the additional services they provide that DOs value. This is illuminated by this case study with a female in-depth interviewee.

### Case study 1 –Esosa (RID29)

Esosa is a 37-year-old female respondent from Gia. Educated to primary school level, she has three children: one boy and two girls, all under 18 years and in school. The family own a mobile phone, a bicycle and a motorbike, farm 5.5 acres but don’t have access to electricity. Esosa laughed when saying that she can’t do everything her husband does when he is away and they have one female donkey with a foal at foot. She thinks donkeys are the most versatile animal she owns, adding that when she ‘did not own a donkey I [had] to borrow [one] from a friend to go to the bush to transport firewood to the house … We know the economic importance of donkeys; that is why we have one in our household. We bought the donkey through the little saving we did by cutting down and skipping our meals at home … As you know, we are no longer healthy enough to carry all these things on our heads … Now we don’t engage in any activity without the use of the donkey, because we depend on it for livelihood … it will be a challenge to my life without it.” Although not used for ploughing, Esosa added that “now that we have our own donkey, is helpful and others too can hire it to perform any activity for a little fee. We would have wished to buy another donkey but now we can’t afford it due to the increase in the price of the donkeys.” Esosa’s children are old enough to work with their donkey, but she is the primary user, transporting firewood from bush to market. “I can go for firewood in the bush for two consecutive days and have enough days to rest and do other chores like cooking for the family and work on my farm … when we are to fetch water with the donkey less time is used as compared to carrying it on our heads. So the donkey can help us save more than two hours […] In fact, this [wet] season [owning a donkey] saves me time to engage in a day job like sowing on someone’s farm for money.” Contributing 60 per cent of Esosa’s total income, her donkey is the main source of her earnings, but she is also a member of her local SUSU [54] because it “is meant for the weak and the poor to save and support the family, and I am weak and poor.”

Esosa’s customers are primarily NDOs who hire donkeys when they need additional power for transportation assistance and ploughing. Those NDOs that cannot afford to hire the services of a donkey and cart are reduced primarily to head loading every domestic and agricultural chore (Table 3). These households survive by using different livelihood strategies to DOs but, as can be seen from the information in Tables 3 and 4, NDOs are well-aware of the benefits donkeys provide in the reduction of daily drudgery. The differences between Esosa and Selma’s daily drudgery are stark.

**Table 4:** Reasons why NDO survey respondents would like to buy a donkey and barriers to donkey ownership (n = 8). Source: Maggs, 2022 [1].

Field study site	RID Number	Reasons	Barriers
Fielmon			
Female	32	To help me in my farming activities and for carrying loads.	Our income level is low (simply we can’t afford its cost).
Female	33	To help with our farming activities and also to carry firewood and water for us. We could also rent it out for cash.	I don’t have the money to buy one because of inadequate income. We depend on other relatives (poverty).
Female	34	To help me in our farming activities, carry firewood, fetch water and other loads. But for now I don’t have the money.	Do not have enough income and also my husband is ill. Hoping the future will be fine for me.
Male	30	I have oxen that I always use to plough. I also use it to plough people’s land and they pay me.	
Male	31	So that it can cart produce, plough, cart water, cart firewood.	I don’t have enough income to buy a donkey.
**Gia**			
Female	36	Because it is very useful: harvests firewood; hire out; carry water; sell vegetables.	I am not working.
Female	37	Because of its economic importance such as harvesting firewood, ploughing etc.	Poverty.
Male	35	Very helpful and it’s a multi-purpose animal.	Can’t afford.

Demand for the services of donkey power has led to the development of a fully-functioning market for the services of donkeys and carts within both study communities. Prices charged for the hire of donkeys varied, with some DOs saying they provide the services of their donkeys free of charge or at reduced rates to friends, family and neighbours, which they might not like to reveal. Kali (RID41), a female in-depth NDO interviewee from Gia has hired or borrowed a donkey to yoke to their cow to plough, or hires bullocks. The family also:

“pay people to plough, sow, and even weed on our farm: we spend around 250GH¢ ($43.33) for the ploughing and we also provided the people who use the cows to plough with food and drinks.” Kali added that livestock/donkey hire disadvantages her because she “must wait for the owner to finish working before giving it to me [and] and it affects my productivity level […] and I will lose whatever amount I would have made to support my household.”

This is probably because she misses the best post-rain window for getting a plough in the ground.

The reasons why NDO respondents aspire to donkey ownership (Table 4) are the same as DOs: for minimising the drudgery in ploughing and transportation, with only one respondent saying they would rent it out for cash. They all cite lack of affordability as the reason they do not own a donkey: the same reason the majority of DOs gave for not owning more than one donkey, like Esosa. This quote, from Abi (RID24), an in-depth interviewee from Fielmon was typical of most female respondents when asked if they would sell their donkey:

“I would never sell my donkeys if they were not very old or sick. Ei!! Sell my donkey and do what? What would I do to feed my family? I don’t have a female child to support me. The donkeys are helping me.”

This data is illustrated by several quotes by male respondents that the donkey “has greater value even than cow/cattle” (MRID3) and: “The cow only works during the wet season! But the donkey is all year around and does a lot of activities” (RID27). The donkey as a multi-purpose animal is just being noted in the literature [30]. Interestingly, a number of adult DOs in this study, described their donkeys as “priceless;” “worth more to me than money” or as “having unmeasurable value.”

### Increasing demand for donkeys

All donkey butchers interviewed were based in Upper East Region. They advised that they have been supplying smoked donkey meat to a market in Kumasi for over fifteen years. Kumasi is Ghana’s second largest city, some 600km south of Navrongo and is the capital of the Ashanti Region. According to the donkey butchers and two additional key informants, demand for donkey meat is increasing in Kumasi, as well as locally. The consumption of donkey meat and intestines in Upper East Region is growing and is now fashionable [31]. As one butcher (RID53) advised:

“It is because the public now knows that donkey meat is wholesome to consume. People are now aware of the profitable nature of the donkey meat trade and a lot of people in Kumasi also consume the meat. This has account for the price increase.”

**Fig 7:**
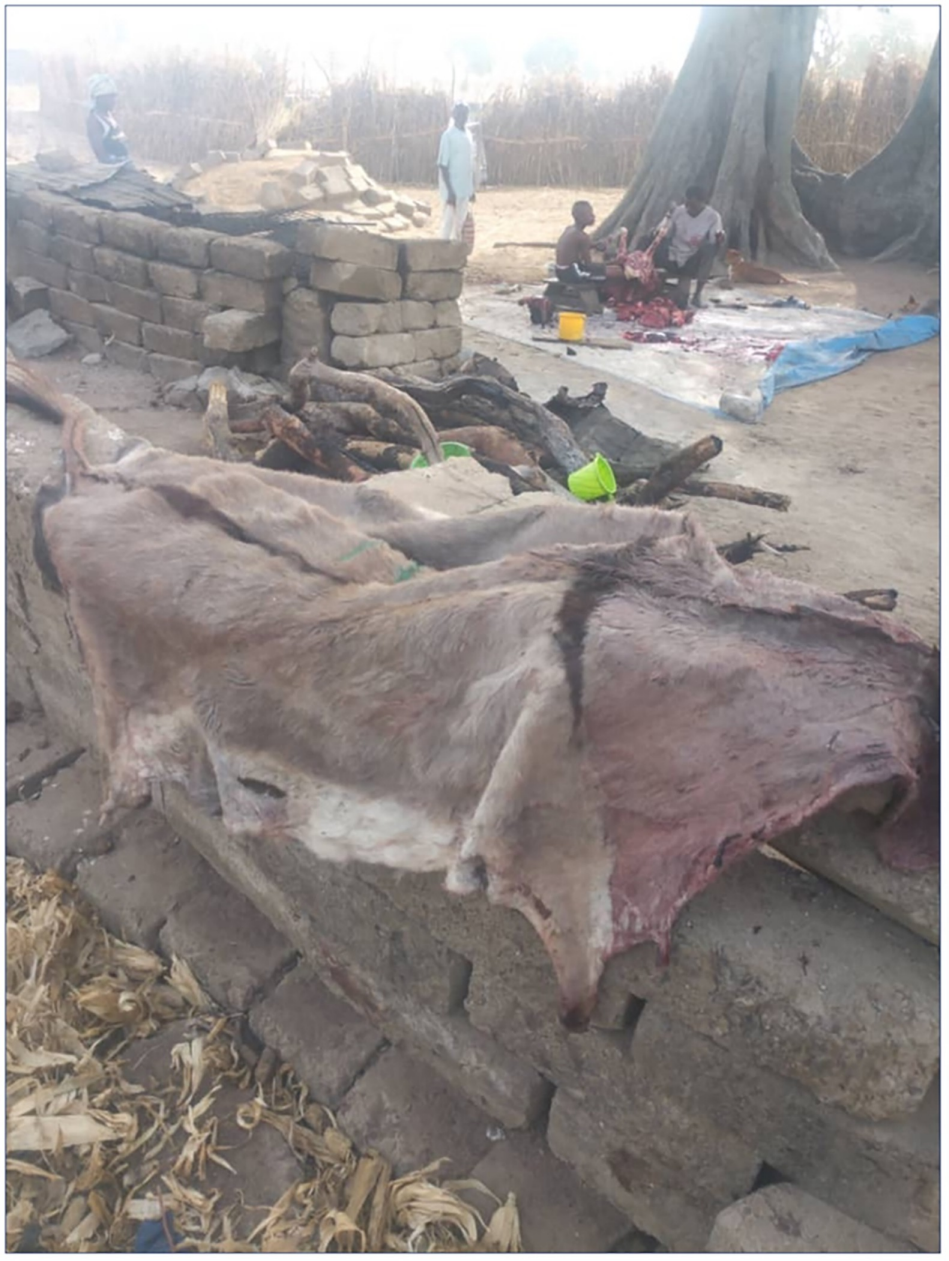
The hide of a recently killed donkey in Gia (UER), to be sold to Chinese buyers, December 2018. Source: © Wepiah, 2019, cc BY 4.0.

The Doba and community donkey butchers advised that a separate market for donkey skins had expanded over the past five years (Fig 7). They reported supplying their surplus donkey hides to middlemen, who may be Chinese or who then sell the skin to “the Chinese:”

“Originally we never wanted the skin of the donkey. The Chinese demanded it to make medicine.” (Doba donkey butcher RID75).

A community donkey butcher reported slaughtering between 10–15 local donkeys twice weekly, so between 1,040 and 1,560 donkeys per year and advised that they buy their “donkeys from Yelwongo in Burkina Faso,” the same market as their competitors. Doba, situated c. eight km East of Navrongo has at least two donkey butchers, who advised that ~8 towns in Upper East Region had butchers providing smoked donkey meat to Kumasi. They informed us that they purchased their donkeys in Burkina Faso, estimating that 100 donkeys a week are slaughtered for meat in Navrongo when demand is high. The donkey butchers were asked whether they only killed old, unproductive donkeys. They answered no, advising that they run businesses and have to supply their customers, so don’t discriminate. “When you get to the market and your supplier comes the ones he brings, those are the ones you buy, whether they are small, young, male, female,” (RID75), although one of them said they don’t kill pregnant or very young donkeys that they can rear (RID76).

When asked if they thought Ghana could run out of donkeys the DBs all said no, because they purchase their donkeys primarily from Burkina Faso, which has many more donkeys than Ghana (Fig 4). This quotation is typical:

“The donkeys we kill are not from Ghana, they are from Burkina Faso and we do not kill Ghanaian donkeys […] The only thing that affects our business is the demand for the meat and the skin. The people we buy from, they purposely rear the donkeys for sale” (RID75).

One key informant, an academic from northern Ghana stated:

“The only objection I have about the trade is the fear that the increasing rate of killing the donkeys might lead to—somehow—near extinction of donkeys because the rate of reproduction is, is quite slow […] and therefore if the current rate continues and it possibly even increases in the demand for the donkey skin and the meat, there is the likelihood for us to be witnessing a near extinction of the donkey.”

However, a significant majority of DOs also do not believe that Ghana could run out of donkeys. Strikingly, the only two survey respondents who agreed that it could were women, with both saying they thought this could happen within five years. Of the ten DO and NDO in-depth interviewees asked, one male DO said yes, five didn’t think so, with two female participants (one DO, one NDO) stating categorically that “Ghana can never run out of donkeys.” Two female DOs were unsure, with one saying:

“How can I know? [Hahahaha] Do I know how long I will live in this world? Mmm … How will I know?” (Maiara, RID28).

**Fig 8:**
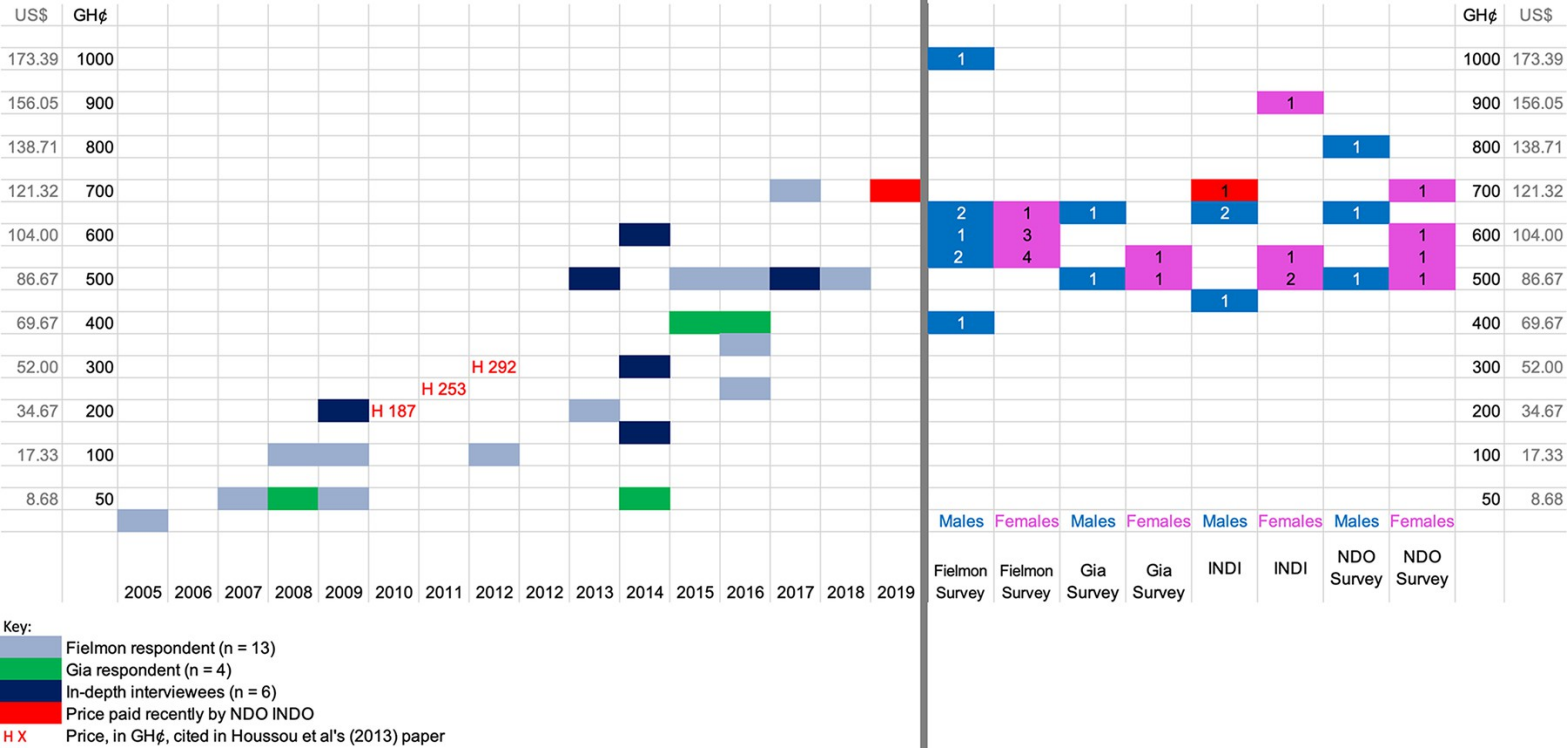
**A:** How much did you pay for your first donkey? When was that? (n = 24) **B:** How much do you have to pay for a donkey now? (n = 34). (Source: Maggs, 2022 [1]).

When asked how much they paid for their donkey (Fig 8A), two female respondents didn’t answer and five females said they couldn’t remember. However, of those who did answer it can be seen that there has been a continual increase in prices since 2005. The data provided is based on individual recall, which cannot always be trusted. The H figures in red text are the mean unit price in GH¢ of donkeys from Houssou et al.’s 2013 field survey [35:5] on animal traction in northern Ghana for comparison purposes. These validate the prices provided by the Fielmon and Gia respondents. All respondents, including all NDOs, advised what they thought the current price of a donkey was (Fig 8B). The price of 750GH¢ ($121.31) marked with a red, in-filled box in both Fig 8A and 8B in 2019, is the amount of money paid by the one NDO in-depth interviewee (RID40), who purchased a donkey between field visits. This price is likely to be highly accurate, as the animal was purchased shortly before the interview.

#### Missing donkeys: Lost or stolen?

Over half of all DO and NDO respondents had personally suffered theft of livestock in the last three years. All DO survey respondents, and the majority of NDOs, said that donkeys did indeed go missing in their community, and all of Gia’s DOs, plus one NDO knew someone whose donkey had gone missing. Five DOs experienced actual theft of their donkey(s), two of whom believe other of their donkeys had got lost as well. The effects of losing a donkey, for whatever reason is illustrated in this quote by Selma, when asked what the impact she had perceived on those who had lost their donkeys (RID38):

“It has affected their work because they were using the donkey to do their work and now they can’t get it to use […] their donkey used to cultivate a particular farm that is very far away from the community prior to the loss but now, they have abandoned that land since they would find it difficult to work effectively without the donkey.”

In Fielmon 13/15 DOs knew someone whose donkey had gone missing, as did all the NDOs: 16 respondents suspected theft, seven that they had got lost and one female and one male DO respondent from Gia said they knew households where some donkeys had both gone missing and others stolen. In answer to the question “Are donkeys going missing in the community?” some male and female in-depth interviewees from both Fielmon and Gia spontaneously replied with the word steal, as well as going missing. These comments were typical:

“They are stealing them so much (RID39); “Yes! They steal them too” (RID27) and “Donkeys are missing so much in the community” (RID25).

An object with no worth is unlikely to be stolen so any animal that is, implicitly has value to the thief. Thus levels of theft are another facet of the value of donkeys. When asked, most said the donkey had probably just got lost, although one respondent said that as his family’s two donkeys were never found they therefore had “been stolen.” A female DO from Gia spontaneously reported that “Not long ago a donkey thief was caught in this community and he was beaten to death,” demonstrating, if true, the depth of community feeling relating to the theft of donkeys. These results have to be placed in the context of theft of livestock in general. As one in-depth interviewee (RID24) said “all animals help us get income,” so all have value, not just as a cash repository. One donkey butcher (RID75) from the Doba community, when asked if any of the donkeys he slaughters could be stolen reported that:

“There are some donkeys that goes missing but whether they are stolen donkeys [we] cannot tell because everything goes missing: fowls, cattle, sheep ….. [laughter].”

This view is supported by a comment by one DO (RID68) girl, who said that her family owned a dog to protect her house, “so that thieves don’t steal [the] donkeys, chicken and goats.”

Most DOs and NDOs corral their livestock at night, in part to facilitate collection of manure for fertiliser. However, DOs face a dilemma with their donkeys. Allowing them to roam increases the probability of their becoming lost or being stolen, but contributes to their receiving free, adequate fodder. Donkeys’ ability to survive in harsh environments results in the “advantage” to DOs that their donkeys can survive without supplementary feeding, thus reducing the cost of ownership, but only if they can graze “for six to seven hours a day on free range” [55:1]. Opinions and the husbandry methods of respondents differed: Maiara (RID28), a 36-year-old female in-depth interviewee from Gia advised that:

“What [I] have to let you know is that when you provide your donkeys with adequate feed they won’t go far away to graze and you will always have it available and you don’t have go round looking for it each day. But when you failed to do that then they will go far away looking for feed and you have to go look them every evening or else they might go missing.”

Caught between additional fodder costs or their donkey going missing, many DOs compromise and release them to roam during the day, with children playing a key role in herding them home in the evening. Fifteen out of twenty-three DOs round them up, tether or confine them in some way overnight; five allowing them to rest and /or feed; one said they left their donkey to roam and two said they brought them near to the house. When asked why he tethered his donkey in their shed Kabira (RID26) said “because someone might steal it.” However, not all DOs who do tether their donkeys at night are concerned solely about the risk of theft, as these two quotes from two female DO in-depth interviewees indicate:

Gia: “We put a rope around its legs to prevent it from roaming at night. We do that because if we free it at night it will destroy someone’s garden and it might be stabbed to death or they will catch it and [demand] you […] pay an amount of 100.00GH¢ ($17.33) before they will release the donkey to you” (RID29).

Fielmon: “I do bring them inside [at night] because if I leave them out they might go and destroy someone’s farm. And if that happens, the farmer owner will summon my husband to the chief’s palace.” (RID25)

**Fig 9:**
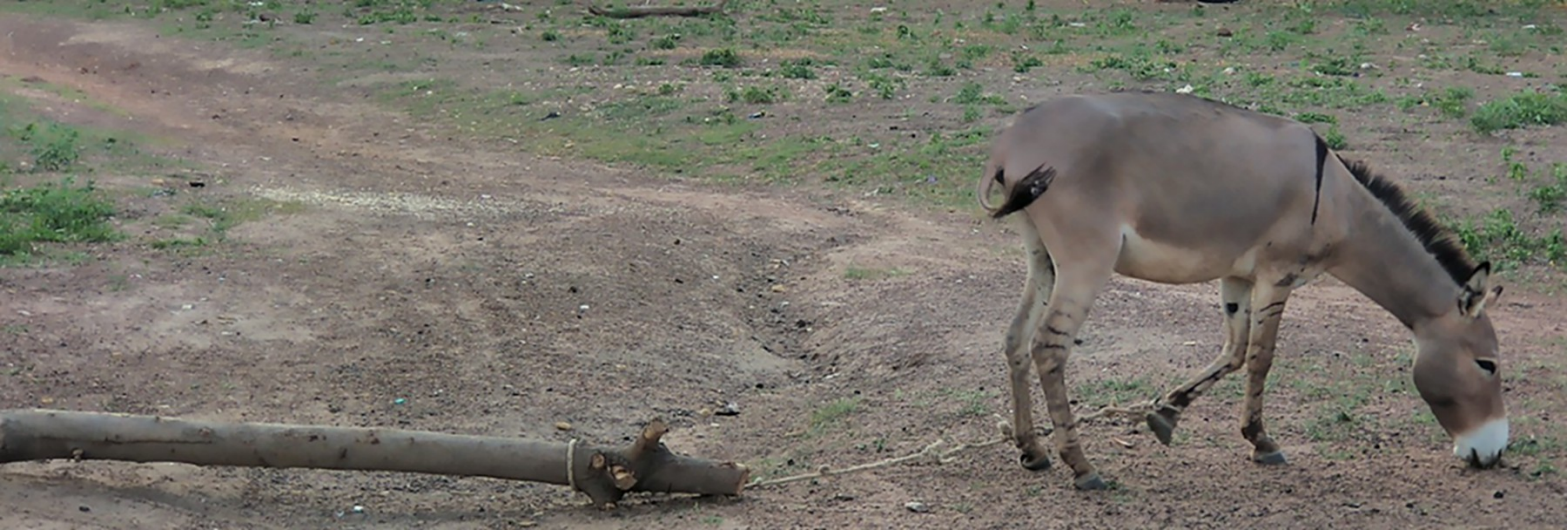
A donkey on a short-term tether in Fielmon. Source: © Maggs, 2019, cc BY 4.0.

DOs employ different strategies to minimise the chance of their donkey getting lost, going missing or being stolen. If it is expected that a family will require their donkey to work during the day then they are tethered close to the house (Fig 9). Most children of both genders in the FGDs said their daily chores include feeding and watering livestock, including their donkeys, illuminated by these quotes: “When I return from school, I eat my lunch, give drinking water to the donkeys [and] retire the donkeys at a greener field…” and “I change their grazing grounds away from the sun.” During field work a number of the welfare assessments undertaken on all donkeys are incomplete because their owner had released their donkey to roam, had no idea where it was and concluded it was too far away to round it up to be evaluated in the time available. One DO’s son released the family’s donkeys just as an assessment was about to commence, and the donkeys took off at speed. Bidden by his mother to retrieve them, this proved impossible.

The obvious difficulty is how far does the donkey roam and whether it remains within the orbit of being found by its owner. All these factors contribute to the problem of missing, lost and stolen donkeys. As noted, one of the unrecognised roles of children in the care and husbandry of donkeys in northern Ghana is to round them up every evening and bring them back to the farm. However, this takes time, depending on how far the donkeys have roamed and obviously reduces the amount of time the child has to undertake other activities, like studying and playing. In her seven daily time budget interviews Jalia (RID25) reported her eldest son “searching for and bringing donkeys back home to sleep” six nights and one morning of the seven. This enables both parents to continue with their daily domestic and agricultural chores with maximum efficiency [31].

### The increasing value of donkeys

**Fig 10:**
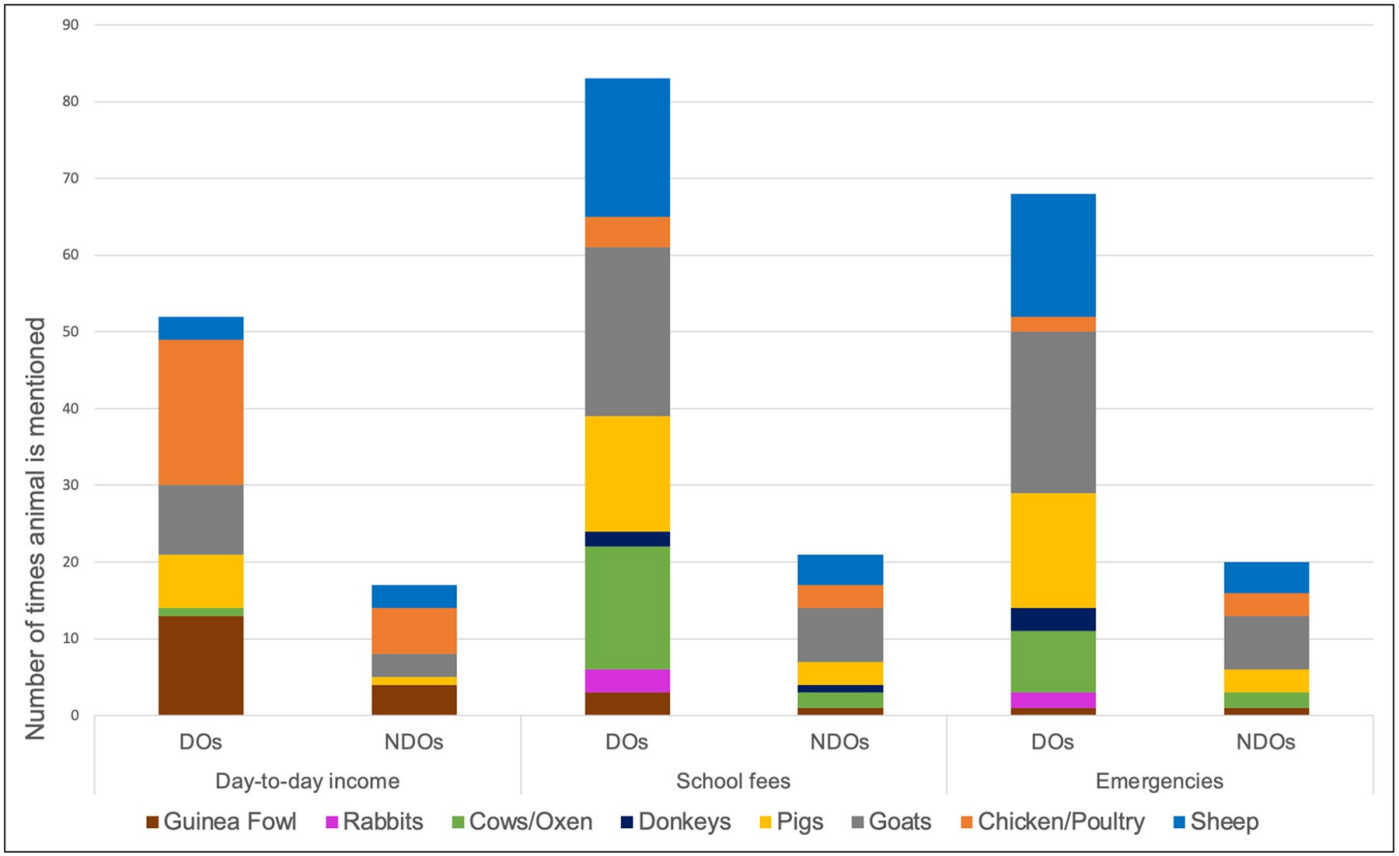
Which animal would you sell first?. Source: Maggs, 2022 [1] Chickens and Other poultry figures combined. Donkey mentioned in NDO school fees column represents the response by the NDO who bought a donkey between field visits.

One of the themes in the donkey literatures prior to the publication of the Under the Skin Report in 2017 [29] was that donkeys had little value [31]. A measure of this lack of value was in comparison to other animals, especially cattle, ownership of which conferred wealth, status and benefits. For example, Yaro (2002) reported that cattle were sold as a last resort [56:12]. Interestingly, donkeys were the least “traded” animals in our study, illustrative of their increasing value as multi-purpose assets. Participants viewed donkeys as the most versatile animal they owned. In a major reversal corresponding with the steady increase in the price of donkeys (Fig 8), far more people said they would be willing to sell their cow or oxen to pay for school fees or in an emergency than they would their donkey (Fig 10). Respondents would sell all other species of animal they owned in most circumstances that they might need sudden cash, saving cattle for major financial outlays: they would only sell their donkey *in extremis*. From the survey data, no-one would sell their donkey to meet day-to-day expenses: this role was primarily taken by smaller animals like chicken, rabbits and guinea fowl. Only one female DO would sell her donkey to pay school fees, compared with twelve who said they would first sell a cow or oxen to do so. In an emergency only two female DOs would sell their donkey in comparison with six who would sell a cow, although a few DO and NDO respondents said it depended on the nature of the emergency. “Middle-sized” animals like sheep, goats and pigs were more likely to be sold to meet school fees or emergencies, with livestock acting, as noted elsewhere, as “walking bank and savings accounts” [57, 58]. NDOs primarily had only smaller animals to sell in emergencies, indicating their ownership of a narrower range of livestock species, with more limited options when faced with unexpected financial demands, in comparison with DOs.

## Discussion

Donkeys in the Majority World are only owned and maintained if worked [42] by people thought of as poor [41]. The more donkeys, the more poor people. The choice of donkeys as the preferred provider of draught power will always depend on dynamic and complicated factors, including cultural tradition, know-how, soils, availability of donkeys and affordable alternatives. Donkeys are the draught animal of preference in both Fielmon and Gia, with the core value they hold being the power they provide in reducing the drudgery of daily domestic and agricultural chores, consistent with the literature. For example, Duncan [59: xv] estimated that in Ghana “women carry up to two head loads of 30kg over an average of 5km a day.” Donkeys also save owners additional costs they would incur on the transportation of various items and time, enabling them to undertake other productive activities, including paid labour.

In agrarian societies like those in northern Ghana it is not surprising that both males and females give ploughing as a core reason for owning a donkey in Fielmon, even by women whose role in the agricultural cycle does not include ploughing. According to a male FGD participant “A woman is not supposed to own a donkey. Men own all the donkeys” (RID78), triangulating the survey data results that all the donkeys were owned by men and managed by women and children. It is likely that these women are talking on behalf of their family’s usage, rather than their own. Further research could investigate the gender issues of donkey ownership in more detail. For example: i) whether the donkeys were bought originally for helping men plough, rather than for the help they provide in reducing the daily drudgery of women and ii) whether a donkey would actually be purchased by a male for his family if it is only needed for female-orientated household chores.

It is interesting to note that some male DOs said they could not farm without a donkey now: at least one female in-depth interviewee said her husband would find it difficult to farm without one either and Esosa referred to not being healthy enough to carry any heavy loads on her head. As an NDO, Selma has no other option but to headload when she cannot afford to hire assistance for her daily chores. There is certainly scope for further research into different aspects of the lives of DOs and NDOs. For example, i) whether donkeys shorten women’s working day and enable increased rest and/or socialising, or whether female DOs work the same length per day as NDO females, but with less drudgery and therefore better health; ii) whether female DOs get more time to market their produce including to what extent they make more money than their NDO sisters. Quantifying exactly how often and how far female NDOs carry building materials, wood, water, pito beer and farm produce through observation and time/motion studies would yield further useful data for comparison with female DOs.

The reported rates of hiring out donkeys for a fee in our questionnaire had an annual frequency of only 2.6%, consistent with that of Tuaruka and Agbolosu’s [53:1] study in Bunkpurugu/Yunyoo District in Northern Region (176km to the East of the Gia study site). The authors concluded that “The least role [for donkey ownership] was for income generation.” However, triangulation across our research instruments indicate that although income-generation appears very much as an after-thought to the reduced drudgery they offer DOs, nearly fifty per cent of owners use their donkeys to make money, the majority of them women. Thus, the ability of a donkey to reduce the drudgery of the chores of both men and women is key to ownership, not income generation. It may be that this benefit is so valuable to DOs that hiring them out for money may be core to respondents’ habitus and only appears as secondary and of less importance to Minority World interpretation. From the DO household’s perspective, optimising the different methods of generating income from the hire of their donkey is complex and layered and typically facilitated by children [30]. DOs may also charge differential rates which they might not want to disclose; there may be an element of response bias or a gendered dimension around the costs of hiring out donkeys which requires further investigation.

A key informant advised that Fielmon have two neighbouring communities in Sissala and Jirapa that consume donkey meat, even though they themselves do not. The increasing fashion for eating donkey meat was mentioned in a focus group of male farmers from Gia (RID53):

R: “At that time, it was believed that eating donkey meat would upset your stomach but now that no longer exists, so people buy them purposely to kill for meat and so their price shot up.”

**Fig 11:**
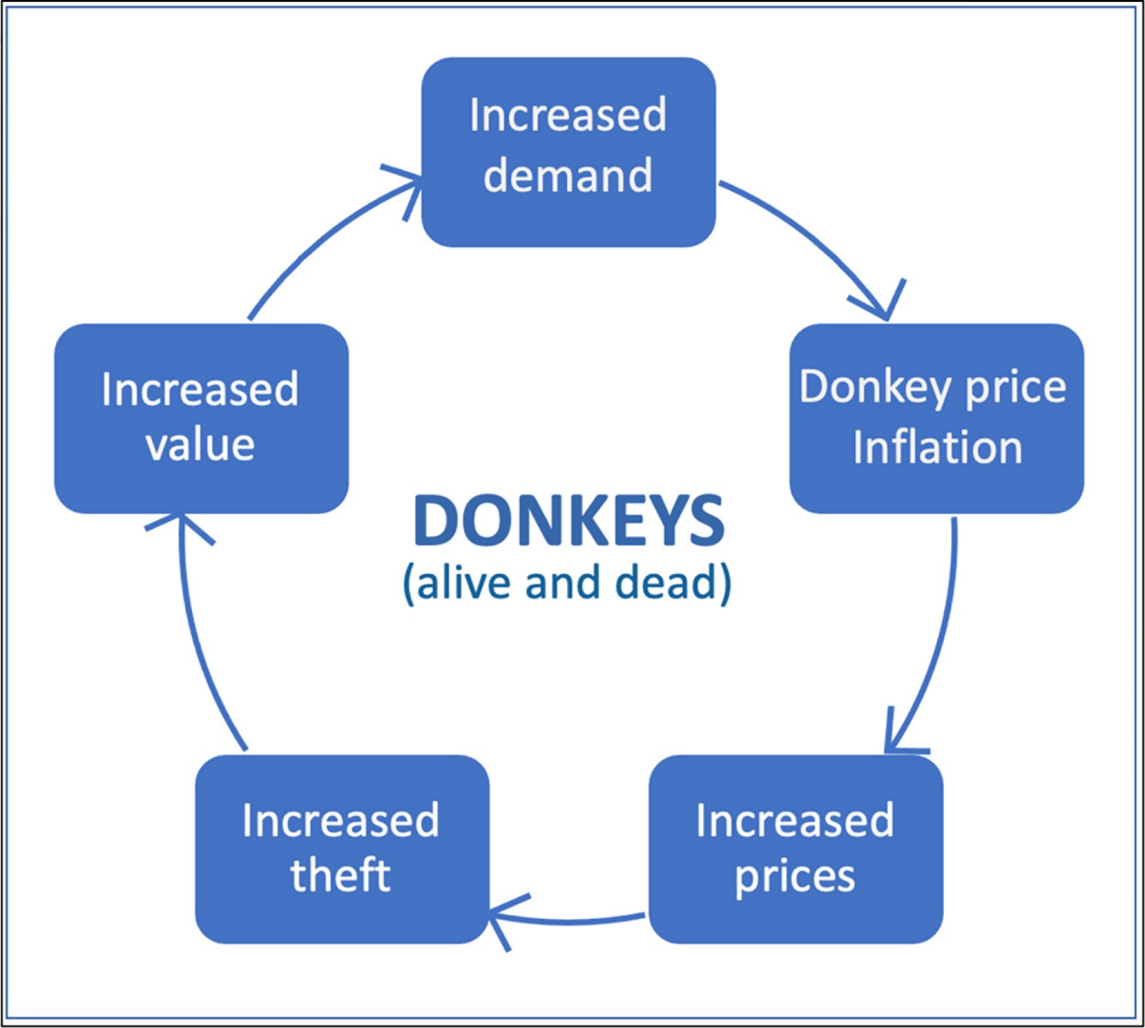
The negative feedback cycle of the market for donkeys in northern Ghana. Source: Maggs, 2022 [1].

Competition between those with different requirements of their donkeys is fuelling demand, which in turn is underpinning the increased prices of donkeys witnessed in Ghana since ~2005. Price and demand are both inextricably linked with the increase in the value of donkeys reported by the study’s participants (Fig 10). Demand for dead donkeys is being driven by both the increasing consumption of donkey meat in northern Ghana, and the donkey hides trade opportunistically riding on the back of this activity, hoovering up unwanted skins. The increasing value of donkeys is based on these intensifying and competing demands for both dead and alive donkeys, seen in the orthodox global economics-orientated diagram below (Fig 11).

Fig 8 demonstrates that donkey prices have increased by ~274% over the past 15 years. In comparison, from a high of 59.32% in 1995, Ghana’s inflation rate in 2005 was 15.1%; it increased to a maximum of 17.46% in 2016 and was 8.73% in 2021 [60]. Prices of donkeys increased even between the six months between field visits: from the broad going rate of 500GH¢ ($104.00) in December 2018 to between 550–750 GH¢,($95.31–121.31) in June 2019. This is in line with the figure of 650GH¢ ($112.64) supplied by a donkey butcher, who is likely to be paying for donkeys more regularly than most of the farmers. However, as one of the community butchers explained, donkey prices vary. A weak, ill or old donkey would cost him “about 400–500 GH¢.” ($69.32–86.65) When asked about how this compares to a young, fit donkey suitable for farming he said:

“It depends on the size of the donkey and whether it is old or young. We can get an old donkey which is big and look very healthy than a younger one. Some farmers might have younger donkeys and do not take proper care of it. In that case it will cost less than the old donkey which is big and healthy. The bigger and fatter it is, the more expensive it will be and the better the profit we make.”

On the figures provided by the donkey butchers, who themselves rely on donkeys for their livelihood, demand for donkey meat is killing an estimated ~11,500–50,000 donkeys per year in the Upper East Region of Ghana (10 slaughterhouses, killing 20–100 donkeys per week, plus the c. 1,000 donkeys killed p.a. by the community butchers). As noted, these figures do not appear to be affecting the donkey population of Ghana, supporting the donkey butchers’ claim that they purchase their donkeys primarily from Burkina Faso (Fig 4).

Given Maiara’s comment that “no one would buy an old donkey that only does eating” (RID28/EARS), it is likely that a high percentage of local donkeys that are old and unproductive are slaughtered, providing meat that is likely to be tough and unappetising [61]. With respondents’ comments that eating donkey meat resulted in upset stomachs in the past it can be hypothesized that: the increasing commercial slaughter of young, productive donkeys, resulting in better-quality meat could be underpinning the rising consumption of donkey meat reported in the Upper East Region and Kumasi. Further studies into this hypothesis are therefore suggested, as better-tasting, good-quality meat may stimulate greater consumer demand: not only within existing donkey-eating communities, but also within those that currently do not eat it. If this happens more pressures will be placed on donkey populations than is currently the case. It seems likely in the absence of formal breeding programmes, coupled with the stable nature of Ghana’s donkey population, that the demand for donkeys for agriculture is met in part from donkeys reproducing naturally, augmented by purchasing replacements from Burkina Faso’s donkey markets. If the demand for live and dead donkeys outstrips supply in both Ghana and/or Burkina Faso as a result of the market trends outlined here, it would be useful to model the implications.

The recent and steep valorisation of donkeys in the post-2017 literature has been generated by the demand for e’jiao and the awareness of this demand in the Minority World. This has placed a spotlight on the value of dead donkeys, but for their skin, rather than their meat. Although this new focus is leading to a re-evaluation of the services donkeys provide, of which this study is a part, the increasing demand for donkey meat has received scant attention [62]. Demand for donkey meat is embedded in northern Ghana, with evidence of a formalised, ~15-plus-year-old market for the product in Ghana’s Upper East Region. This is seeing increasing demand in two key markets: locally and in Kumasi. Recognition of donkeys as multi-purpose assets by farmers contributes to driving up demand: farmers only sell their donkeys *in extremis* or at the end of their productive life, indicating their recognition of the lifetime value of their donkeys. The need for donkeys by this sector of the market competes with demand for donkeys for meat, which also drives increases in the animals’ price (Fig 8). According to the DO and NDO respondents in both Upper East and Upper West Regions, this process has been on-going since at least ~2005. It is therefore unlikely to have been initiated by the demand for donkey hides alone.

There is also evidence that in the last four to six years, elements of the donkey hide trade are opportunistically riding on the back of the pre-existing and growing donkey meat market in the Upper East Region. This is a synergistic relationship, with donkey hides purchased as a waste product of the meat trade. The price of donkeys has been increasing above the rate of inflation since at least 2007, before the arrival of the e’jiao trade’s middlemen in ~2014. We argue therefore that the key drivers of this price rise must be the increasing demand for donkeys for use in both agricultural households and for meat. More research on these topics needs to be undertaken to enable the links between donkey populations, donkey hides/e’jiao and donkey meat to be more clearly understood. It would also be valuable to investigate: i) the effect the donkey hides trade is having on the price and numbers of donkey in Burkina Faso; ii) whether the traders in Burkina Faso that the Doba donkey butchers purchase their donkeys from do actually “purposely rear the donkeys for sale” (RID75) and iii) whether donkey owning communities in other countries are also now selling their cattle before their donkey and only selling their donkeys in an emergency. If this is the case the reasons why should be researched as well, to see whether the value/price of donkeys has overtaken the value of cows, because DOs now view donkeys as more difficult to replace than cattle for example.

Implicitly indicative of value, trying to safely secure one’s donkey is not just to protect its welfare in preventing it from getting lost or being stolen. All respondents are aware of the threat to their livelihood if they lose their donkey(s), describing their lives as “going backwards” should this occur. All the donkeys of two Fielmon families died during an haemorrhagic pneumonia epidemic between field visits. Neither household could afford to replace them by the date of their second interview in June 2019, joining the ranks of NDOs “to be pitied” as one DO male in a FGD described them. How farmers try to keep their donkeys safe results from an intricate mix of factors involving their nutrition, natural tendency to roam, individual farmer resource and social norms. When 13-year-old Gloria (RID61) was asked what her roles and responsibilities were with regard to her donkey she replied, “I lead it to feed on weeds.”

Despite the wry smile Gloria’s answer engenders, problems could arise if the donkey hasn’t been able to browse sufficient fodder even for its maintenance during a 24-hour cycle. Supplementary feeding may also be needed for additional nutritional requirements during pregnancy, lactation, and growth [63, 64]. These are outside the scope of this paper but “There is much less information on [feeding] donkeys than there is on horses” [63:1] and it is likely that their diets are deficient, especially if tethered all night without access to adequate amounts of fodder. Thus, even before roaming to the point of getting stolen or lost, (although the donkey may know exactly where it is), the ways that donkeys are kept ironically predispose them to an increased risk of going missing, getting lost or being stolen. This results in a number of negative impacts on their welfare, which can include painfully-applied identification marks, tethering, sub-optimal nutrition and theft for the donkey meat and/or skin trades. Hard-pressed DOs are therefore caught between allowing the animals to roam or finding the money to purchase additional feed, which few appear to do.

**Fig 12:**
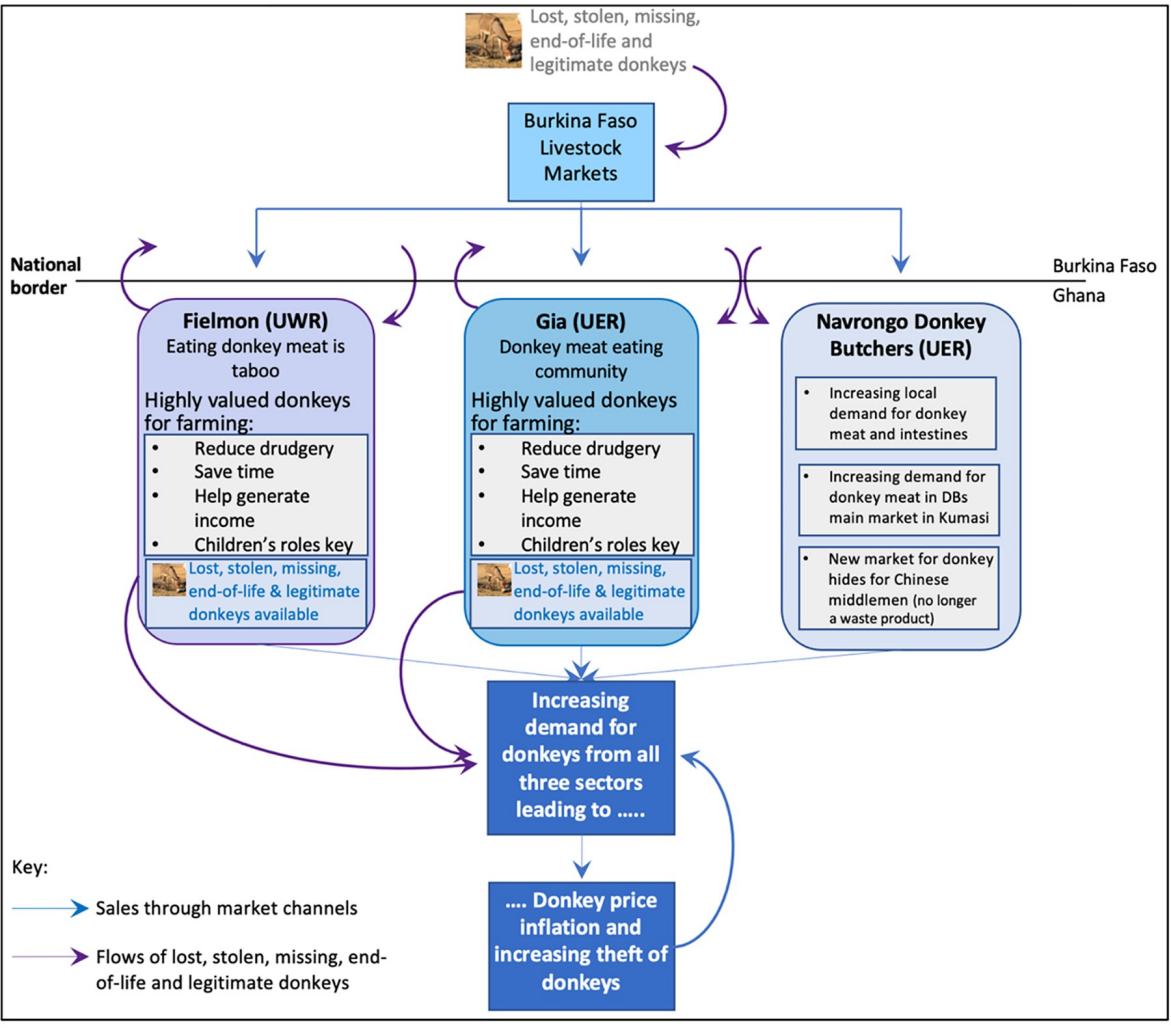
Overview of demand and price/value cycle of donkeys in northern Ghana. Donkeys may go missing and get lost or stolen from their original home; they may be stolen directly or may be sold at livestock markets by their original owner for various legitimate reasons including being at the end of their productive life. Source: Maggs, 2022 [1].

Links between husbandry norms, roaming behaviour, lost/missing donkeys, theft and the meat trade are relational and inextricably complex. Despite DOs’ best efforts to secure their donkeys, an unknowable percentage of the animals from both communities go missing, get lost or are stolen. They are never returned to their rightful owners, despite the branding and skin mutilations practised to aid identification. It is likely that many of these donkeys either end up being slaughtered for the meat and hides trade before the end of their useful, productive life or with a new owner. This is beneficial for the new owner but may be better or worse for the donkey. Husbandry methods and financial constraints therefore contribute to the increasing cost and value of donkeys, both in communities that do, and do not, eat donkey meat. This in turn contributes to the demand, price, value cycle of donkeys, linking donkeys that are lost, stolen, missing and/or owned legitimately, including those at the end of their productive life into an on-going feedback cycle. Thus, both the use-value and exchange-value of donkeys are adjusting to the changing donkey market environment on the ground. Therefore, the generic economic diagram (Fig 11) can be developed for northern Ghana specifically (Fig 12). The closeness to the much larger population of donkeys in Burkina Faso supports the meat and hides trades in northern Ghana. This is important, given that the donkey butchers report that they are killing somewhere between just under the total official number of Ghana’s donkeys per annum (~11,000 vs ~15,000), to over three times the official number of donkeys(~15,000 vs ~50,000), whilst Ghana’s population of donkeys appears to stay relatively stable (Fig 4).

The services donkeys provide are highly valued within the farming communities of Fielmon and Gia. There is increasing demand for the animals, summarised by this discussion within a male focus group (RID78):

R6: “As it was mentioned, if you pick a hoe to plough, you can’t plough up to the size that would have been ploughed by the donkey. So, the donkeys help you to farm faster so that you can plant early. None of the other animals apart from bullocks can farm. The donkeys still help women to carry their firewood home and carry water home. Bullocks can farm, but they can’t carry water nor other load. This is why the donkey’s work is much appreciated and valuable.”

R7: “In terms of pricing, cattle have higher values but the donkeys are still more valuable than the cattle.”

A substantial majority of farming respondents in Fielmon believe donkeys are expensive to purchase and that the price of a donkey had increased since they had bought theirs (91%, n = 23), with eight DOs saying they had always been expensive. Many respondents said prices are high due to increasing demand, effectively limiting their ability to purchase more because donkeys are “now seen as an asset” and a multi-purpose animal:

“You see, donkeys are not like other animals where its price falls and rises. Donkeys’ price keeps on increasing day by day because of its usefulness” Maiara (RID28).

As noted, NDOs are well-aware of the benefits donkeys provide in the reduction of daily drudgery. The added advantages of saved time, increased opportunities to generate additional income and the health and well-being advantages the use of donkeys offer are all visible to NDOs. Further research could provide additional information and insights, potentially quantified, into the differences donkeys make to people lives, including, for example whether NDOs own a smaller range of productive species, with less numbers of each type of livestock. Given comments made about differences in yields by NDOs, equally pertinent research questions to examine include whether NDOs suffer smaller yields and therefore greater food insecurity and hunger than DOs. How Selma undertakes her daily chores (Table 3) clearly illuminates why DOs are so focused on owning donkeys for the reduction in their daily domestic and agricultural drudgery. Sadly, the poorest NDOs may never be able to afford a donkey, especially given the increasing prices donkeys now command: just surviving for these families is difficult enough. As Kali (RID41) advised:

“The produce gotten from the farm is not even sufficient to feed the family not to even talk of selling the produce to buy a donkey.”

## Conclusion

This research provides a timely and necessary study into the substantial value live donkeys have to poor farming households in northern Ghana. However, it also highlights as well that dead donkeys hold substantial value to other poor people trying to make their living: donkey butchers. It is likely that donkeys will always be valued for their extrinsic, rather than their intrinsic worth: the interpretation of a donkey’s value to an increasing range of actors will vary, but is predominantly utilitarian in nature.

For those who cannot afford an alternative on farm, the value of donkeys to their owners is their assistance in reducing the drudgery and saving time of everyday tasks. Primarily, these chores are for ploughing and other farming activities, and for domestic, marketing and agricultural transport. Hiring out donkeys to generate income is an important secondary role for those who own donkeys, especially women but is not as important to DOs as the help they give in reducing back-breaking drudgery. Most NDOs would like to own a donkey for these reasons but cannot afford one, especially as their cost keeps increasing well above inflation. Potential actions that could help farmers in this situation would be to: i) increase awareness of both the much-needed help donkeys provide smallholders and how valuable their owners perceive them to be; ii) increase awareness that donkeys often cannot be easily substituted by mechanised power; iii) make it easier to obtain a loan or micro finance to purchase a donkey than other things, given their ability to help generate income for hard-pressed families and iv) involve local charities if possible in innovative and creative ways to integrate donkeys into their service offerings.

The perception of donkeys in northern Ghana is superseding the ‘under-valued and invisible’ narrative of the donkey in “the literatures.” Donkeys are valued as “priceless” and perceived as multi-purpose animals; are preferred for ploughing over cattle, achieving near-equivalent prices to them and are now worth stealing. Sadly, the way donkeys are currently kept and husbanded predisposes them to increasing levels of loss and theft, which in turn compromises their welfare.

However, for donkey butchers the value of the animals lies in the profit per carcase and skin, the burgeoning donkey meat market providing a number of butchers in northern Ghana with their livelihoods from dead donkeys. To maximise their livelihood, donkey butchers obviously need to maximise the number of donkeys they slaughter. Only time will tell whether the increase in the consumption of donkey meat reported by this study is a short-term fashion limited to Ghana, a permanent trend limited to Ghana or a permanent international trend. Because of the increase in in the value of donkeys to all these actors however, donkey populations are under threat, leading to a negative demand, price, value, demand feedback cycle for donkey services (Fig 12). The people who rely on the services of either live or dead donkeys in northern Ghana are being thrust from their normally low level of incorporation in the global economy into the developing international markets for donkey hides and growing markets for donkey meat. These markets are significantly increasing the competition for donkeys, as well as their price.

Donkeys sit within this complex nexus of drivers. International working equid NGOs are raising awareness of the threat to donkey populations to governments to overcome donkeys’ historical invisibility [31]. The attendant geopolitical and illegal trade implications of the donkey hide trade to donkey populations are unpredictable and fast-paced [65]. That the threat to donkey populations has been recognised by Ghana’s government is implicit in the regulations they have put in place to reduce the slaughter of Ghanaian donkeys. These regulations may be having a positive impact on the numbers of donkeys in the country, given Ghana’s officially stable, long-term donkey population. However, the closeness of the national border to Burkina Faso—and its porousness—makes it difficult to offer solutions, especially as the donkey hides trade is not, in and of itself, illegal and governance issues often make enforcing any regulations difficult. The greatest threat appears to be to the donkey population of Burkina Faso. Key to the long-term survival of donkeys—in Ghana, their neighbours and globally—is the hope that e’jiao consumers could one day accept the product grown in the laboratory, from donkey stem cells [66]. The value of donkeys outlined in this paper are relevant to these global issues and could contribute to increasing awareness of the threat to donkey populations, policy debates about donkeys, and a broader recognition of their significant contribution to rural livelihoods in Majority World countries.
